# Error Analysis of Clay-Rock Water Content Estimation with Broadband High-Frequency Electromagnetic Sensors—Air Gap Effect

**DOI:** 10.3390/s16040554

**Published:** 2016-04-18

**Authors:** Thierry Bore, Norman Wagner, Sylvie Delepine Lesoille, Frederic Taillade, Gonzague Six, Franck Daout, Dominique Placko

**Affiliations:** 1 SATIE, ENS Cachan, CNRS, Paris-Saclay University, Cachan 94230, France; fdaout@u-paris10.fr (F.D.); dominique.placko@satie.ens-cachan.fr (D.P.); 2 Institute of Materials Research and Testing at the Bauhaus-University, Weimar 99423, Germany; norman.wagner@mfpa.de; 3 French National Radioactive Waste Management Agency (Andra), Chatenay-Malabry 92298, France; Sylvie.Lesoille@andra.fr; 4 Simultation & Information Technologies for Power Generation Systems Department, EDF, Chatou 78400, France; frederic.taillade@edf.fr; 5 LISIS COSYS, Ifsttar, University Paris Est, Champs sur Marne 77477, France; gonzague.six@ifsttar.fr

**Keywords:** water content measurement, TDR probe, clay-rock, dielectric spectroscopy, frequency domain finite element modeling

## Abstract

Broadband electromagnetic frequency or time domain sensor techniques present high potential for quantitative water content monitoring in porous media. Prior to *in situ* application, the impact of the relationship between the broadband electromagnetic properties of the porous material (clay-rock) and the water content on the frequency or time domain sensor response is required. For this purpose, dielectric properties of intact clay rock samples experimental determined in the frequency range from 1 MHz to 10 GHz were used as input data in 3-D numerical frequency domain finite element field calculations to model the one port broadband frequency or time domain transfer function for a three rods based sensor embedded in the clay-rock. The sensor response in terms of the reflection factor was analyzed in time domain with classical travel time analysis in combination with an empirical model according to Topp equation, as well as the theoretical Lichtenecker and Rother model (LRM) to estimate the volumetric water content. The mixture equation considering the appropriate porosity of the investigated material provide a practical and efficient approach for water content estimation based on classical travel time analysis with the onset-method. The inflection method is not recommended for water content estimation in electrical dispersive and absorptive material. Moreover, the results clearly indicate that effects due to coupling of the sensor to the material cannot be neglected. Coupling problems caused by an air gap lead to dramatic effects on water content estimation, even for submillimeter gaps. Thus, the quantitative determination of the *in situ* water content requires careful sensor installation in order to reach a perfect probe clay rock coupling.

## 1. Introduction

The interaction of electromagnetic waves with matter is sensitive to various physical and chemical parameters ([1,2]). Hence, high frequency (radio and microwave) electromagnetic methods (HF-EM) present a strong potential for quantitative estimation for water content for porous media in geoenvironmental engineering [3]. Moreover, HF-EM method gives the opportunity to quantitatively explore porous material in both laboratory or field scale investigations with non or less invasive sensors [3]. Laboratory scale allows broadband measurements under controlled boundary (humidity, temperature, pressure …) conditions, whereas field scale allows long time non-invasive or minimally invasive monitoring of structure. The transfer of the knowledge determined in controlled laboratory measurements to the field scale requires the development of robust sensors. Time or frequency domain reflectometry (TDR [4] or FDR [5]) methods fulfill this requirement for quantitative estimation of spatial and temporal evolution of the water content in porous media.

In this context, the HF-EM method represents a powerful tool for the safety solutions of management for nuclear structure or nuclear waste repository. The objective of the French National Radioactive Waste Management agency (Andra) is to collect and manage long term the radioactive waste produced in France. For high-level and long-lived radioactive waste, the solution of deep geological disposal was selected [6]. In 2005, the feasibility of such disposal was concluded; an industrial center, called CIGEO, is now studied. Electricité de France (EDF) is in charge with nuclear power plants in France, which include numerous concrete buildings (containment vessels and reinforced concrete natural draft for example). Water content in nuclear structures and nuclear waste repositories (concrete and/or clay-rock) is an important parameter to understand in order to predict the behaviour along the lifecycle and at the end of the operating period [7,8]. On the one hand, the underground installations of the Cigeo repository will be built progressively and should operate over a period on the order of a century [9] and on the other hand, the average operation time of nuclear power plants is currently about 30 years, and they are expected to still provide electricity for 15 to 40 years. In this context, Andra and EDF are involved in research programs dedicated to remote monitoring of Thermo-Hydro-Mechanical (THM) processes. *In situ* water content assessment technologies is an important challenge of this joint program.

**Figure 1:**
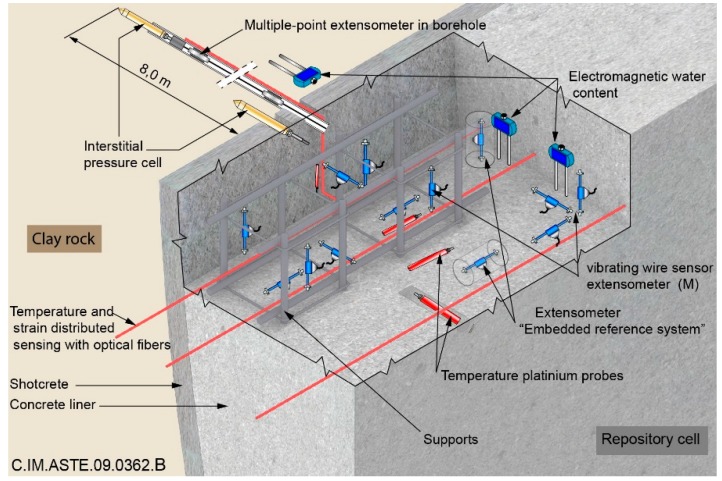
Envisioned monitoring in a nuclear waste repository.

Figure 1 illustrates the envisioned instrumentation of one repository cell. Sensors would be implemented in the concrete liner of repository cells to supply a global characterization of the evolution of the structures. Extensometers, such as vibrating wire sensors (WVS) in concrete, inductive measurements in boreholes and interstitial pressure cells (IPC), would provide the mechanical evolution; platinium probes are qualified to provide temperature measurements. Punctual measurements would be completed with distributed optical fibers sensors (OFS). In this framework, we focus here on the application of T/FDR method for water content measurement of the Callovo-Oxfordian (COx) clay rock.

Despite the efficiency of such a method, it actually provides indirect information about water content. The main difficulty concerns the estimation of water content from the performed permittivity measurement. The first solution consists of using empirical models. Numerous models exists for soil [10,11], unfortunately not valid for clays. Another possibility is to develop laboratory calibrations for each materials [12,13]. However, laboratory calibration encounters opposite specifications: (i) the need for large samples to place the TDR probes and respect interaction field; (ii) duration of water content forced variations. Hence, the broadband dielectric characterization of non-disturbed clay rock as a function of water content in laboratory scale is necessary. These aspects were addressed in the first part of this study, based on the previous experimental investigations according to [14].

Another challenge concerns the implementation of the probe in the host material and the bias between modeling and reality. Numerous study consider an idealized equivalent circuit and do not take into account losses due to the skin-effect or radiation from the sensor [15,16]. For these reasons, the spatial sensor characteristic of a commercial TDR probe (Campbell Scientific [17]) was numerical analyzed with broadband electromagnetic finite element modeling technique with Ansys High Frequency Structure Simulator (HFSS [18]). To achieve the most reliable computations possible, the measured frequency dependent effective complex permittivity were considered in the constitutive material equations. Additionally, an important issue was the practical method to insert T/FDR probes into hard clay-rock. The coupling between the rods of the probes and the surrounding medium remains an important feature for field applications. Different types of probes were implemented in the Andra URL, but a perfect coupling between sensors and clay rock could not be reach. In this configuration, the air gap could lead to drastic under or over estimation of water content. The impact of submillimetric air gap on frequency and time reflection coefficient was computed with our modeling. Classical travel time analysis (on TDR waveform) in combination with the frequently used empirical Topp equation [12] and the theoretical Lichtenecker and Rother model (LRM see [19,20] for details) were applied to quantify the error on water content estimation. These aspects will be presented in the second part of the paper.

## 2. Electromagnetic Properties of Clay Rock

### 2.1. Materials

The investigated COx clay rock samples are clay-rich materials that contain up to 50% clay (illite and illite/smectites mixed layer), carbonates and tectosilicates (quartz) [21]. The porosity of COx varies between 0.1 and 0.2. The pores diameter distribution ranges between nanometer and hundreds of nanometer [22]: they are mostly distributed between clay particles and within the interlayer space of swelling clay minerals (e.g., smectite).

A non-destructive two port coaxial transmission line was used for this study. For hard material, it requires that the material under test be carefully drilled and shaped so as to avoid gaps between the material and the cell. The complete procedure of samples preparation is described elsewhere [23]. Just note that samples were stored at defined relative humidity (ϕ) in climatic chambers to impose their degree of saturation S_w_. The porosity n and the degree of saturation S_w_ were measured after the electromagnetic characterization. To that purpose, several measurements were performed on each sample. First, samples were dried in order to measure their water content (θ) and their dry density (*ρ*_d_). 150 °C dehydration in nitrogen atmosphere is preferred to 105 °C, because the highly structured water at clay interface and within the interlayer space cannot be fully extracted at 105 °C. In this study, we consider five samples. See Table 1 for the hydro-mechanical characterization of the samples.

### 2.2. Broadband Dielectric Measurements

**Figure 2:**
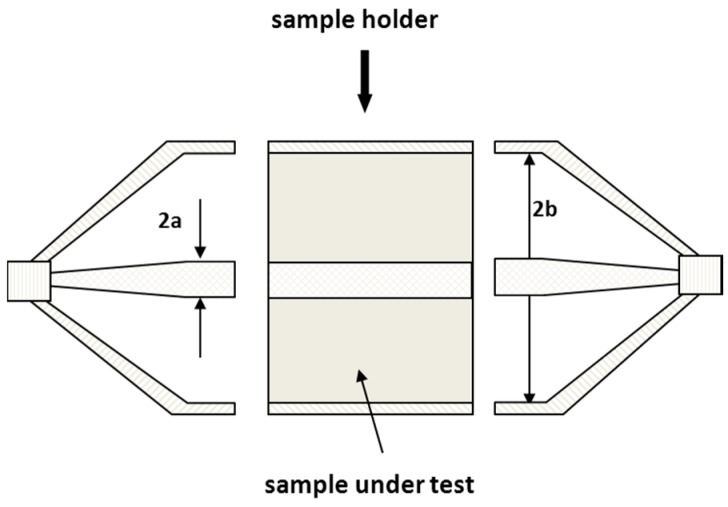
Scheme of the used coaxial cell for material analysis.

The dielectric spectrum were experimental determined over the 1 MHz to 10 GHz frequency range with network analyser technique. The cell consists of three sections: two transitions units and a sample holder (see Figure 2 and [24] for a complete description of the cell). The dimensions of the cell are: inner radii a = 6.52 mm, outer radii b = 15 mm, sample holder length 20 mm long. The coaxial cell length is finally 152 mm long.

In general, assuming propagation of the electromagnetic wave in the cell in Transverse Electrical and Magnetical (TEM) mode and non-magnetic materials ($\mu_{r,eff}^{*}=1$), relative effective complex permittivity $\varepsilon_{r,eff}^{*}$ of a sample in the coaxial transmission line is related to its complex impedance $Z_{S}^{*}$ or complex propagation factor $\gamma_{S}^{*}$ as follows [25]:

(1) $$\varepsilon_{r,eff}^{*}={\left(\frac{Z_{0}}{Z_{S}^{*}}\right)}^{2},\varepsilon_{r,eff}^{*}={\left(\frac{c_{0}\gamma_{S}^{*}}{j\omega }\right)}^{2}\;or\;\varepsilon_{r,eff}^{*}=\frac{c_{0}Z_{0}}{j\omega }\left(\frac{\gamma_{S}^{*}}{Z_{S}^{*}}\right)$$

Herein, *Z*_0_ is the characteristic impedance of the empty transmission line, *c*_0_ = (ε_0_μ_0_)^−0.5^ the velocity of light with ε_0_ the absolute dielectric permittivity, μ_0_ the absolute magnetic permeability of vacuum, ω = 2π*f* the angular frequency. For solving the set of equations in (1) based on measured S-parameters a robust and efficient iterative inversion approach according to Barker-Jarvis *et al.* [26] (BJI) was used (see [14] for details).

For the validation of the iterative approach and decomposition of the underling polarization mechanisms inverse modeling technique based on the numerical calculation of the S-parameters with a forward model (TEM-based) of the used coaxial transmission line cell in combination with a generalized dielectric relaxation model (GDR, [27]) was applied. This model assumes three relaxation processes and apparent direct current conductivity:

(2) $$\varepsilon_{r,eff}^{*}-\varepsilon_{\infty }=\frac{\Delta \varepsilon_{\alpha }}{1+j\omega \tau_{\alpha }}+\frac{\Delta \varepsilon_{\alpha \prime }}{1+{\left(j\omega \tau_{\alpha \prime }\right)}^{a_{\alpha \prime }}}+\frac{\Delta \varepsilon_{\beta }}{1+{\left(j\omega \tau_{\beta }\right)}^{a_{\beta }}}-j\frac{\sigma_{DC}}{\omega \varepsilon_{0}}$$

where τ_i_ stands for relaxation time, Δε_i_ for relaxation strength, a_i_ for the stretching coefficient for the *i*-th process and σ_DC_ the apparent direct current electrical conductivity. Please note that the α process corresponds to the main free water relaxation process. Thus, the stretching coefficient was set to 1.

### 2.3. Analysis of Dielectric Spectra

**Figure 3:**
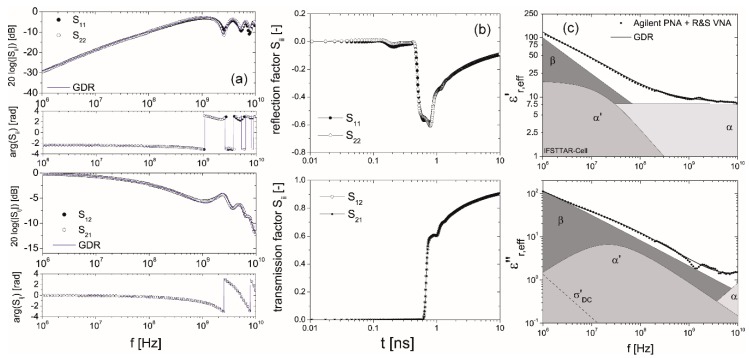
(**a**) S-Parameter S_ij_; (**b**) TDR waveform; and (**c**) complex effective relative permittivity $\varepsilon_{r,\mathrm{eff}}^{*}$ as a function of frequency f of a sample at intermediate water saturation (θ = 0.066 m^3^/m^3^, *n* = 0.14 and S_W_ = 0.46) as well as the results of the parameterization with a global optimization procedure.

For the determination of the relaxation behavior the full set of measured S-parameters was parameterized based on a TEM forward model in combination with Equation (2) assuming three relaxation processes (Figure 3, for details see [14]). A global optimization approach according to Vrugt *et al.* ([28], Shuffled complex evolution Metropolis algorithm—SCEM-UA) was used to parameterize the spectra. This algorithm is an adaptive evolutionary Monte Carlo Markov chain method [29]. The frequency dependence of the electromagnetic properties obtained with the BJI and the result of the inverse modeling technique for a sample at an intermediate water saturation is represented in Figure 3.

**Figure 4:**
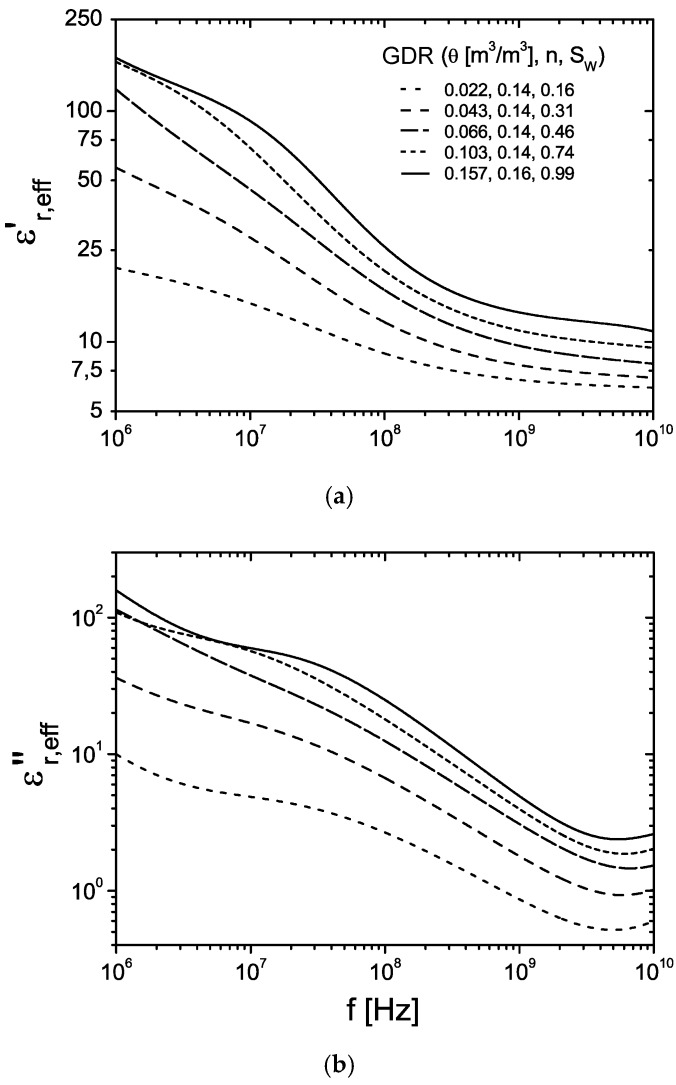
Dielectric input spectra of the clay rock for the numerical 3-D calculation as a function of frequency at defined states determined based on inverse modeling technique with the GDR. (**a**) Real part (**b**) Imaginary part.

In Table 1, the dielectric relaxation parameters of the analyzed data sets are compiled. The complex effective permittivity determined with inverse modeling technique as a function of frequency for the samples at the different defined states relevant for the *in situ* application are represented in Figure 4. The systematic increase of the static permittivity as well as relaxation time of the α process indicates the presence of free pore water. The strong α’ processes with relaxation times between 7.6 and 20.8 ns overlap the water signal (see Figure 3). The low frequency range is dominated by a broad β relaxation process attributed to a distribution of Maxwell-Wagner effects superimposed with low-frequency counter ion relaxation effects with a broad distribution of relaxation times >1 μs.

Hence, it is expected that the apparent permittivity obtained with classical broadband TDR techniques contains not only the water-content contribution but also effects due to water-mineral interaction processes.

## 3. 3D Numerical FEM Simulations

### 3.1. Sensor Configuration

**Figure 5:**
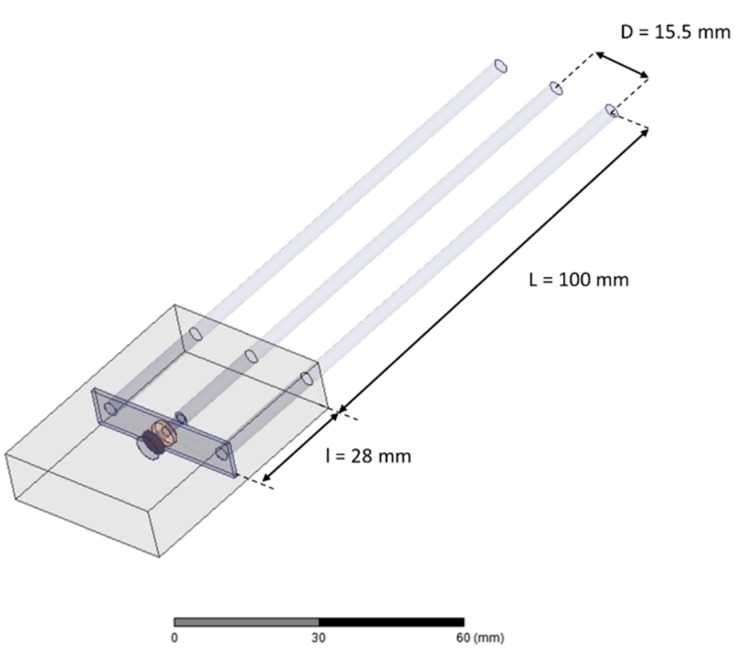
Probe geometry.

The scattering function S11(ω) and step response of a commercial three rods TDR probe (CS 630/CS 635—Campbell Scientific, Inc., Logan, UT, USA) was numerical determined by means of 3-D frequency domain finite element modeling with Ansys HFSS (see [30] for details). For ease of modeling, we consider here a 10 cm long probe which is shorter than the original probe with 15 cm. The other dimensions are identical to the real probe dimensions: 15.5 mm mutual rod spacing and 3.2 mm outer rod diameter. In the model iron rods were used. The transition between rods and coaxial feeding line was made of copper. For the head of the probe Sanoprene with a relative permittivity of 2.3 was used. The dimensions are 5.7 × 4.0 × 1.25 cm. We further used a short 3 mm coaxial feeding line. In Figure 5 the model structure of the probe is shown. The objective here is not to study the whole TDR/FDR set up (e.g., a long coaxial cable and the probe) but to model the behavior of the probe as realistic as possible by taking into account radiation effects and electrical losses due to the skin effect. Moreover, the dielectric relaxation spectra of the COx presented in Figure 4 were used as input for the 3-D numerical modeling. All around the material and the probe, an air box was used to apply a boundary radiation condition approximation (not represented on Figure 5). The accuracy of this boundary depends on the distance between the boundary and the object from the radiation emanates. A sensitivity analysis was carried on this distance using standard material (air, methanol, water) and electrical lossy materials (such as clay rock).

The numerical calculations were performed on an unstructured mesh generated with a λ/3 based adaptive mesh refinement at 3 GHz. The broadband complex S_11_(ω) function was calculated with an interpolating sweep at frequencies in the range from 10 MHz to 3 GHz. The corresponding TDR waveform was computed based on an input voltage step with 300 ps rise time.

To focus on the practical problem of inserting the probe in the hard clay rock, an air gap was added between the rods and the material. We consider an air cylinder surrounding each rod. In this configuration, the value of the gap, called e_gap_, is defined as the distance between the rod and the clay rock. A parametrical study on e_gap_ was performed to quantify its impact on time and frequency signals. In our study, e_gap_ ranges from 50 µm up to 1 mm.

### 3.2. Electric Field Distribution

**Figure 6:**
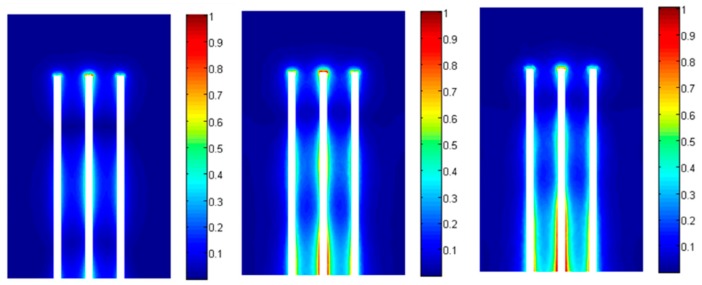
Electric field distribution @1 GHz in the YOZ plane (from left to right: θ = 0.022 m^3^/m^3^, θ = 0.066 m^3^/m^3^ and θ = 0.157 m^3^/m^3^).

**Figure 7:**
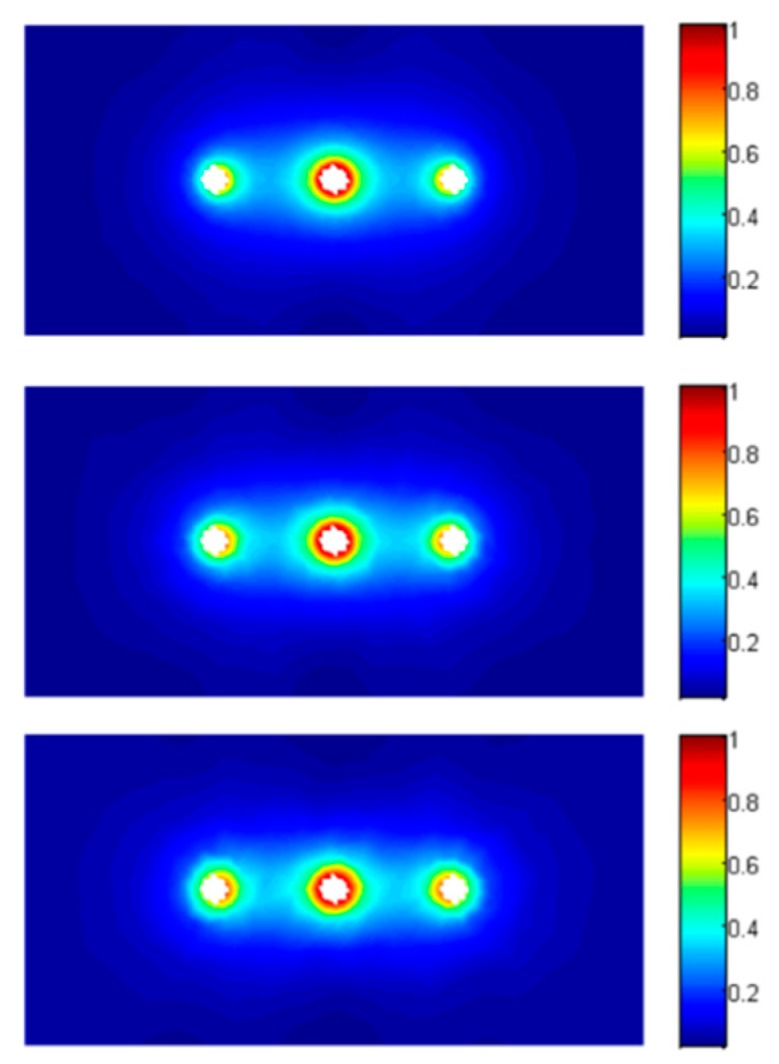
Electric field distribution @1 GHz in the XOY plane in the middle of the rods (from top to bottom: θ = 0.022 m^3^/m^3^, θ = 0.066 m^3^/m^3^ and θ = 0.157 m^3^/m^3^).

In Figure 6, the normalized complex magnitude of the electric field E_n_ = |**E**|/|Emax| in the YOZ plane is represented for 3 different types of clay rock sample presented in section II (θ = 0.022 m^3^/m^3^, θ = 0.066 m^3^/m^3^ and θ = 0.157 m^3^/m^3^) at 1 GHz in the case of perfect contact. The same quantity is plotted in a XOY plane in the middle of the rod on Figure 7.

Qualitatively, the numerical simulations show that the sensitivity changes along the probe (Figure 6). The electric field in a cross section of the rods (Figure 7) shows that most part of the energy is located in near vicinity of the rods. This means that any local non uniformities around the rods will have a great effect on the performed permittivity measurement. For a hard material such as clay rock this implies an important challenge for inserting the rods. The operation of drilling holes must be realized carefully: if not the permittivity measurement could be strongly affected by any cracks along the probe.

### 3.3. Frequency and Time Signals

**Figure 8:**
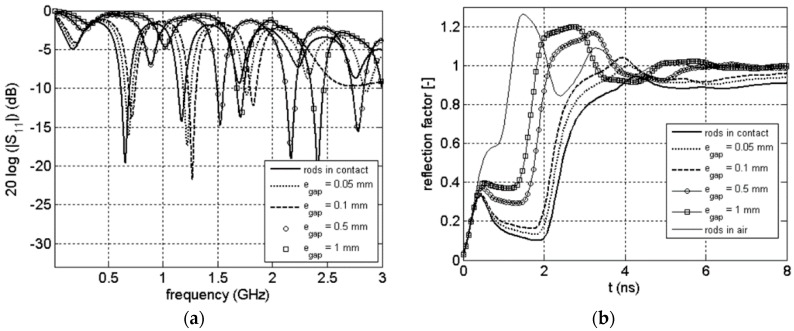
Magnitude of scattering function S_11_(ω) (**a**), corresponding TDR waveform (**b**) for the probe surrounded by air and clay rock (θ = 0.022 m^3^/m^3^).

**Figure 9:**
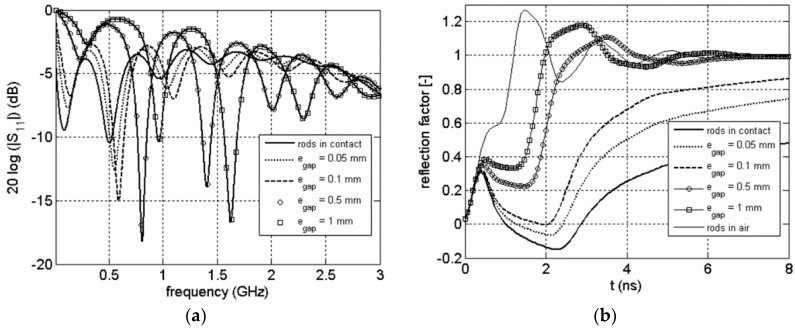
Magnitude of scattering function S_11_(ω) (**a**), corresponding TDR waveform (**b**) for the probe surrounded by air and clay rock (θ = 0.066 m^3^/m^3^).

**Figure 10:**
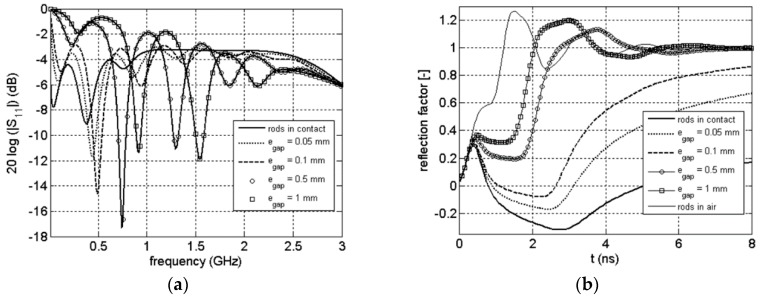
Magnitude of scattering function S_11_(ω) (**a**), corresponding TDR waveform (**b**) for the probe surrounded by air and clay rock (θ = 0.157 m^3^/m^3^).

In Figure 8, the magnitude of the S_11_(ω) scattering function as well as the corresponding TDR is plotted for the commercial probe surrounded by air and clay rock (θ = 0.022 m^3^/m^3^) for different value of the thickness of the air gap. As a reference, the frequency response of the probe when perfect contact is added on the figure. For the time domain, we added another reference: the case when the probe is completely surrounded by air. The Figure 9 and Figure 10 show the same results for a clay rock sample defined by θ = 0.066 m^3^/m^3^ and θ = 0.157 m^3^/m^3^ respectively.

Coupling problems caused by air gap lead to dramatic effect even for very small gaps. The signals in frequency domain show a shift of the resonances to higher frequencies. The attenuation of the signals, which is increasing with water content in the case of perfect contact between rods and clay rock [31], is decreasing with the increase of the thickness of the air gap. The air gap effect is more easily observable for TDR wave form. We can observe a systematic decrease of the two way travel time of the signal reflected from the probe end as well as a decrease of the absorption with increase of the thickness of the air gap. All those comments have to be linked with the electric field distribution. As explained before, during a time or frequency measurement only the material in near vicinity of the rods is involved. An air gap along the rods will act as a quasi-waveguide: the influence of the surrounding medium is then strongly reduces (even for submillimeter gap).

### 3.4. Classical Travel Time Analysis

Classical travel time analysis was applied to the obtained TDR signals based on the dual tangent method to find the onset as well as inflection point of the total reflected signal to compute apparent relative permittivity ε_app_ ([4,32]). The onset point corresponds to the intersection of the tangent lines whereas the inflection point corresponds to the apex of the derivative. This analysis was performed for the whole set of TDR curve (e.g., for the five clay rock samples) to quantify the error of travel time analysis due to air gap.

Table 2 gives the apparent permittivity obtained with onset method for perfect contact and for some values of the air gap. For each value the relative deviation between expected (e.g., perfect contact) and obtained values is given. As a reference, the value obtained at 1 GHz for ε’_r,eff_ is shown. Table 3 compiled the same results obtained with inflection method.

The values obtained when the probes are in perfect contact gives the first information. Classical travel time analysis underestimates or overestimates the value of the permittivity at 1 GHz. As pointed out in Section 2, this is related to dispersion and absorption due to effects by water—clay mineral interaction. This clearly indicates that the apparent permittivity contains not only the water-content contribution, but also valuable information due to water-mineral interaction processes which are mostly disturb the unambiguous quantitative determination of the volume fraction of free pore water. This comment can be linked with the results from [33]. A method to compute the effective measurement frequency of TDR waveform was developed. The study has shown that the effective frequency for dispersive and conductive media is expected to be below 0.6 GHz.

The presence of air gap between the rods and the material leads to drastic under estimation of the apparent permittivity for both method. For example with onset method, an air gap of 0.05 mm gives a relative error of 0.09 on the apparent permittivity for a low water content (θ = 0.022 m^3^/m^3^), a relative error of 0.12 for an intermediate water content (θ = 0.066 m^3^/m^3^) and a relative error of 0.17 for a high water content (θ = 0.157 m^3^/m^3^). The errors obtained with inflection method are lightly higher.

The water content was estimated from the apparent permittivity obtained from travel time analysis using Topp equation (Equation (3)—[9]) and Lichteneker and Rother theoretical mixing model (LRM, Equations (4) and (5)—[19,20]):

(3) $$\theta_{Topp}=-5.3\times 10^{-2}+2.92\times 10^{-2}\varepsilon_{app}-5.5\times 10^{-4}{\varepsilon_{app}}^{2}+4.3\times 10^{-6}{\varepsilon_{app}}^{3}$$

(4) $$\varepsilon_{app}^{\alpha }=\theta \cdot \varepsilon_{w}^{\alpha }+(1-n)\varepsilon_{G}^{\alpha }+(n-\theta )$$

which yields:

(5) $$\theta_{LRM}=\frac{\varepsilon_{app}^{\alpha }-(1-n)\varepsilon_{G}^{\alpha }-n}{\varepsilon_{w}^{\alpha }-1}$$

In Equations (4) and (5), ε_w_ represents the permittivity of water phase whereas ε_G_ represents the permittivity of solid particles and α the structure parameter. In this application, the values of ε_w_ was set to 80 , the values of ε_G_ was set to 5.55 according to [14]. For the structure parameter, the value was adjusted to obtain the best fit: a value of 0.75 was found. LRM is frequently used with α = 0.5 and is then called CRIM [34].

**Figure 11:**
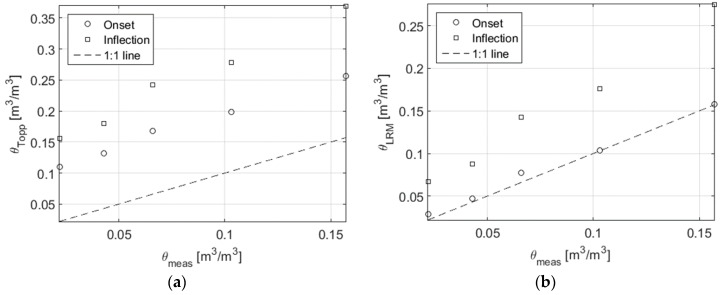
Estimated water content with Topp equation (**a**) and LRM (**b**) computed with apparent permittivity obtained by onset and inflection method for the rods in contact.

In Figure 11, the estimated water content with Topp equation and LRM was plotted against the measured water content (the values obtained by gravimetric method, see Table 1) for the rods in perfect contact for onset and inflection method.

Topp method strongly overestimates water. The results obtained with LRM and Onset method gives a decant estimation of water content (see Table 4 for the relative error on water content determination). As we could expect, mixture equation (LRM) is more accurate than Topp equation since it takes into account the rock structure trough porosity.

The error in the determination of water content with LRM and onset method in the presence of the air gap are compiled in Table 4. The results show a dramatic error on the estimation. For example, for a realistic air gap of 0.1 μm the relative error ranges from 0.34 to 1.05.

## 4. Conclusions

The dielectric relaxation behavior of clay-rock from a sedimentary rock formation at the Underground Research Laboratory in Bure (Meuse, France) was studied in the radio and microwave frequency range (1 MHz–10 GHz) under atmospheric conditions with the coaxial transmission line method in a previous study [26]. Samples were conditioned at defined relative humidity to achieve water saturation between 0.16 to nearly saturation. The relaxation behavior was quantified based on a generalized fractional relaxation model under consideration of an apparent direct current conductivity assuming three relaxation processes: a high-frequency water process and two interfacial processes (see [26] for details). These two processes are related to interactions between the aqueous pore solution and mineral particles (adsorbed/hydrated water relaxation, counter ion relaxation and Maxwell-Wagner effects).

The measured broadband electromagnetic material properties of COx were used as input data in 3-D FEM numerical calculations of a commercial available three rod TDR probe. This approach allows a realistic modeling of the FDR or TDR response of the probe since it takes into account: (i) intrinsic dispersion and absorption of the material; (ii) radiation effects of the probe and (iii) electrical losses of the metal parts due to the skin effect. The electric field distribution, the scattering function and TDR waveform of the probe embedded in the clay rock at different states in terms of water content, water potential and porosity were computed. To focus on the problem of probe insertion in such a hard material, an air gap was added between the probe and the clay rock.

In the case of perfect contact, the field distribution has shown qualitatively the spacial sensitivity of the probe. Most part of the energy is located in near vicinity of the rods with a change of the special sensitivity along the sensor due to field pattern. Coupling problems caused by air gap lead to dramatic effect even for very small gaps for the frequency and time domain signals. To quantify the air gap effect, classical travel time analysis was applied on TDR waveform. The frequently used empirical Topp equation and the theoretical mixture equation according to Lichetenecker and Rother (LRM) was used to estimate water content from apparent permittivity. The mixture equation considering the appropriate porosity of the investigated material provide a practical and efficient approach for water content estimation. The results clearly indicate that the effects due to coupling of the sensor to the material cannot be neglected in case of quantifying the water content of the material. Therefore, an appropriate approach is needed which enable the identification of the correct coupling between sensor and material. We urgently need to to focus on the practical and efficient method to insert TDR probes into hard clay rock. The frequency dependence can be included in such approaches, which will be the next step in our analysis.

## Figures and Tables

**Table 1:** Relaxation parameters of Equation (2) used in the modeling of the effective relative permittivity $\varepsilon_{r,eff}^{*}(\omega ,\theta ,\varphi ,n,S_{W})$ of the clay rock needed for the FEM modeling for defined states in terms of relative humidity ϕ, volumetric water content θ, porosity n and water saturation S_W_ (taken from [14]).

ϕ (%)	θ (m^3^/m^3^)	n (-)	S_W_ (-)	ε_S_ (-)	ε_∞_ (-)	Δε_α_ (-)	τ_α_ (ps)	Δε_α’_ (-)	τ_α’_(ns)	1–a_α’_ (-)	Δε_β_ (-)	τ_β_ (μs)	1–a_β_ (-)	σ_DC_ (μs/m)
11.1	0.022	0.136	0.160	6.21	1.50	4.72	1.35	13.2	10.1	0.383	789	74.4	0.27	0.08
33.0	0.043	0.140	0.306	6.81	1.48	5.33	1.83	36.2	17.5	0.353	1069	30.0	0.33	0.05
57.7	0.066	0.144	0.458	7.71	1.73	5.98	2.33	20.1	7.6	0.234	1312	5.3	0.40	0.05
75.3	0.103	0.139	0.740	9.29	4.11	5.18	4.35	129.9	20.8	0.276	1148	25.1	0.27	0.06
99.9	0.157	0.158	0.991	11.06	8.19	2.87	7.77	172.1	20.0	0.31	24,115	90.9	0.005	4.6

**Table 2:** Onset method. Comparison between apparent permittivity for the perfect contact configuration and real part of the effective permittivity at 1 GHz. Apparent permittivity obtained for different values of the air gap, values in brackets are the relative error between expected (e.g., perfect contact) and obtained values.

	Perfect Contact		Presence off the Air Gap					
	ε^‘^_f,eff_ @ 1 GHz	ε_app_	e_gap_ = 0.05 mm ε_app_	e_gap_ = 0.1 mm ε_app_	e_gap_ = 0.2 mm ε_app_	e_gap_ = 0.3 mm ε_app_	e_gap_ = 0.4 mm ε_app_	e_gap_ = 0.5 mm ε_app_
air	-	1.037	-	-	-	-	-	-
clay rock θ = 0.022	6.843	6.280	5.707 (0.09)	4.769 (0.24)	4.160 (0.34)	3.650 (0.42)	3.306 (0.47)	3.023 (0.52)
clay rock θ = 0.043	7.907	7.270	6.529 (0.10)	5.929 (0.18)	4.608 (0.34)	4.039 (0.44)	3.591 (0.50)	3.245 (0.55)
clay rock θ = 0.066	9.613	8.966	7.890 (0.12)	7.027 (0.22)	5.832 (0.35)	4.574 (0.49)	4.016 (0.55)	3.589 (0.60)
clay rock θ = 0.103	11.04	10.533	8.989 (0.15)	7.925 (0.25)	6.429 (0.39)	5.423 (0.48)	4.320 (0.59)	3.811 (0.64)
clay rock θ = 0.157	14.04	13.814	11.390 (0.17)	9.626 (0.30)	7.461 (0.46)	6.125 (0.56)	5.240 (0.62)	4.191 (0.70)

**Table 3:** Inflection method. Comparison between apparent permittivity for the perfect contact configuration and real part of the effective permittivity at 1 GHz. Apparent permittivity obtained for different values of the air gap, values in brackets are the relative error between expected (e.g., perfect contact) and obtained values.

	Perfect Contact		Presence off the Air Gap					
	ε^‘^_f,eff_ @ 1 GHz	ε_app_	e_gap_ = 0.05 mm ε_app_	e_gap_ = 0.1 mm ε_app_	e_gap_ = 0.2 mm ε_app_	e_gap_ = 0.3 mm ε_app_	e_gap_ = 0.4 mm ε_app_	e_gap_ = 0.5 mm ε_app_
air	-	1.623	-	-	-	-	-	-
clay rock θ = 0.022	6.843	8.397	7.828 (0.06)	6.750 (0.20)	5.751 (0.31)	5.282 (0.37)	4.833 (0.42)	4.403 (0.47)
clay rock θ = 0.043	7.907	9.595	8.986 (0.06)	8.397 (0.12)	6.750 (0.30)	5.751 (0.40)	5.282 (0.45)	4.833 (0.50)
clay rock θ = 0.066	9.613	12.94	10.873 (0.16)	9.595 (0.26)	8.397 (0.35)	6.750 (0.48)	5.751 (0.55)	5.282 (0.59)
clay rock θ = 0.103	11.04	15.187	12.231 (0.19)	10.873 (0.28)	8.986 (0.41)	7.828 (0.48)	6.240 (0.59)	5.751 (0.62)
clay rock θ = 0.157	14.04	22.056	15.975 (0.27)	13.669 (0.38)	10.224 (0.54)	8.986 (0.59)	7.828 (0.64)	6.240 (0.71)

**Table 4:** Relative error on water content estimation with Onset method and LRM.

	Perfect Contact	Presence off the Air Gap					
		e_gap_ = 0.05 mm	e_gap_ = 0.1 mm	e_gap_ = 0.2 mm	e_gap_ = 0.3 mm	e_gap_ = 0.4 mm	e_gap_ = 0.5 mm
θ = 0.022	0.294	0.205	1.052	1.626	2.125	2.472	2.765
θ = 0.043	0.099	0.219	0.484	1.093	1.370	1.596	1.775
θ = 0.066	0.176	0.109	0.345	0.686	1.065	1.242	1.382
θ = 0.103	0.004	0.248	0.428	0.693	0.881	1.098	1.203
θ = 0.157	0.002	0.240	0.425	0.665	0.823	0.932	1.069
